# Supplementary material for: Under pressure—exploring partner changes, physiological responses and telomere dynamics in northern gannets across varying breeding conditions
Source: PeerJ. 2023 Dec 1;11:e16457. doi: 10.7717/peerj.16457 (PMC10695113; doi:10.7717/peerj.16457)
Supplement: Supplemental Information 1 — The three first models and null model shown were derived from the following initial equation: biomarker (1—ID) + Partnership status × Year + Partnership status × Breeding stage + Sex. We calculated an Akaike information criterion value corrected for small sample (AICc), and we selected the most parsimonious model including partnership status (first objective of the study) with the lowest AICc. Model including partnership status with unreasonable Δ AICc (> 2.5) were excluded. Yr, year; PartnStatAft, partnership status between year 1 and year 2; BrSta, breeding stage; diff, difference in biomarker results between year 1 and year 2; z, standardized data; l, log-transformed data; r: residuals used; TL, relative telomere length (T/S ratio); TROC, annual telomere rate of change; OHdG, plasma 8-hydroxy-2’-deoxyguanosine concentration; TBARSt, plasma thiobarbituric acid reactive substances divided by plasma triglycerides concentration; TAC, total antioxidant capacity of plasma; PIc, peroxidation index in blood cells; PIp, peroxidation index in plasma; ω 6/ω 3c, omega-6/omega-3 ratio in blood cells; ω 6/ω 3p, omega-6/omega-3 ratio in plasma; BM, body mass; BMvar: body mass variation during the breeding season; HL, heterophils:lymphocytes ratio; HCT/TP, hematocrit divided by plasma total protein concentration; GLU, plasma glucose concentration; TRIG, plasma triglycerides concentration; BHB, plasma beta-hydroxybutyrate concentration; TP, plasma total protein concentration; ALB, plasma albumin concentration; GLOB, plasma globulin concentration; A/G, albumin/globulin ratio in plasma; URIC, plasma uric acid concentration; CK, plasma creatine kinase activity. [file peerj-11-16457-s001.pdf]

TABLE S1. Summary of the first linear mixed models computed for annual differences calculated for each biomarker studied (log-transformed and standardized). The three first models and null model shown were derived from the following initial equation: biomarker ~ (1|ID) + Partnership status × Year + Partnership status × Breeding stage + Sex. We calculated an Akaike information criterion value corrected for small sample (AICc), and we selected the most parsimonious model including partnership status (first objective of the study) with the lowest AICc. Model including partnership status with unreasonable ΔAICc (> 2.5) were excluded.

Biomarkers	The three first models and null model (with only ID as a random parameter)	AICc	ΔAICc	Weight
zl TL	1|ID + Yr	245.4	0.00	0.339
	1|ID + Yr + BrSta	247.0	1.31	0.151
	**1|ID + Yr + PartnStatAft (selected model)**	**247.3**	**1.97**	**0.127**
	1|ID	286.2	40.83	0.000
zl TROC	1|ID + Yr + BrSta	155.5	0.00	0.324
	1|ID + Yr	157.2	1.64	0.143
	**1|ID + Yr + BrSta + PartnStatAft (selected model)**	**158.0**	**2.49**	**0.093**
	1|ID	174.0	18.49	0.000
zl OHdG diff	1|ID + Yr	139.8	0.00	0.255
	1|ID	141.0	1.27	0.135
	1|ID + Yr + BrSta	141.4	1.69	0.110
	**1|ID + Yr + PartnStatAft (selected model)**	**141.8**	**2.07**	**0.091**
zl $TBARS_t$ diff	1|ID	163.3	0.00	0.162
	1|ID + BrSta	163.4	0.08	0.156
	**1|ID + BrSta + PartnStatAft + BrSta*PartnStatAft (selected model)**	**164.1**	**0.76**	**0.111**
	1|ID + Yr	165.0	1.66	0.071
zl TAC diff	**1|ID + Yr + PartnStatAft + URIC (selected model)**	**128.1**	**0.00**	**0.365**
	1|ID + Yr + PartnStatAft + Sex + URIC	130.5	2.39	0.110
	1|ID + Yr*PartnStatAft + PartnStatAft + URIC	130.8	2.64	0.098
	1|ID	152.4	24.22	0.000
zl PI_c diff	**1|ID (selected model)**	**129.8**	**0.00**	**0.311**
	1|ID + Sex	131.7	1.91	0.120
	1|ID + Yr	131.8	2.08	0.110
	1|ID + BrSta	132.1	2.36	0.095
zl PI_p diff	**1|ID + Yr + PartnStatAft + BrSta (selected model)**	**-37.6**	**0.00**	**0.927**
	1|ID + Yr + PartnStatAft + BrSta + PartnStat*BrSta	-31.3	6.31	0.039
	1|ID + Yr + PartnStatAft + BrSta + PartnStat*Yr	-30.5	7.12	0.026
	1|ID	52.6	10.95	0.004
zl $\omega6/\omega3_c$ diff	1|ID + Yr + Sex	128.0	0.00	0.281
	1|ID + Yr	128.3	0.26	0.247
	**1|ID + Yr + PartnStatAft (selected model)**	**130.2**	**2.14**	**0.096**
	1|ID	136.2	8.18	0.005

Table S1. Continued

Biomarkers	The three first models and null model (with only ID as a random parameter)	AICc	ΔAICc	Weight
zl ω6/ω3$_p$ diff	**1|ID (selected model)**	**62.3**	**0.00**	**0.285**
	1|ID + Yr + BrSta	63.9	1.63	0.126
	1|ID + BrSta	64.0	1.71	0.121
	1|ID + Sex	64.2	1.90	0.110
zl HL diff	1|ID + Yr	179.4	0.00	0.358
	1|ID + Yr + Sex	181.8	2.37	0.109
	**1|ID + Yr + PartnStatAft (selected model)**	**181.8**	**2.40**	**0.108**
	1|ID	183.0	3.60	0.059
zl HCT/TP diff	**1|ID + Yr + BrSta + PartnStatAft (selected model)**	**163.4**	**0.00**	**0.293**
	1|ID + Yr + BrSta	164.2	0.82	0.194
	1|ID + Yr + BrSta + PartnStatAft + PartnStatAft*Yr	165.6	2.23	0.096
	1|ID	182.2	18.77	0.000
rzl BM diff	**1|ID + BrSta + PartnStatAft (selected model)**	**133.2**	**0.00**	**0.280**
	1|ID + BrSta	134.4	1.21	0.153
	1|ID + BrSta + PartnStatAft + Sex	135.6	2.47	0.081
	1|ID	135.7	2.57	0.077
rzl BMvar diff	**1|ID + Yr + PartnStatAft (selected model)**	**106.8**	**0.00**	**0.353**
	1|ID + Yr + PartnStatAft + BrSta + PartnStatAft*BrSta	108.7	1.88	0.138
	1|ID + Yr + PartnStatAft + PartnStatAft*Yr	109.2	2.37	0.108
	1|ID	109.4	2.63	0.095
zl GLU diff	1|ID + Yr + BrSta	171.2	0.00	0.233
	**1|ID + Yr + BrSta + PartnStatAft (selected model)**	**172.3**	**1.12**	**0.133**
	1|ID + Yr + BrSta + Sex	172.3	1.12	0.133
	1|ID	186.3	15.12	0.000
rzl TRIG diff	1|ID + BrSta	151.4	0.00	0.238
	**1|ID + BrSta + PartnStatAft (selected model)**	**152.5**	**1.07**	**0.140**
	1|ID + BrSta + Yr	153.1	1.67	0.103
	1|ID	154.4	2.99	0.053
zl BHB diff	**1|ID + BrSta + Yr (selected model)**	**114.9**	**0.00**	**0.170**
	1|ID	115.3	0.33	0.144
	1|ID + BrSta	115.7	0.78	0.115
	1|ID + Yr	116.3	1.34	0.087
zl TP diff	**1|ID + Yr + BrSta + PartnStatAft (selected model)**	**163.1**	**0.00**	**0.219**
	1|ID + Yr + PartnStatAft	164.1	0.98	0.134
	1|ID + Yr + BrSta + PartnStatAft + PartnStatAft*Yr	164.5	1.41	0.108
	1|ID	178.5	15.39	0.000

Table S1. Continued

Biomarkers	The three first models and null model (with only ID as a random parameter)	AICc	ΔAICc	Weight
zl ALB diff	**1|ID + Yr + PartnStatAft (selected model)**	**164.4**	**0.00**	**0.155**
	1|ID + Yr	164.7	0.23	0.138
	1|ID + Yr + PartnStatAft + PartnStatAft*Yr	165.6	1.11	0.089
	1|ID	168.4	3.99	0.021
zl GLOB diff	**1|ID + Yr + BrSta + PartnStatAft (selected model)**	**165.1**	**0.00**	**0.229**
	1|ID + Yr + PartnStatAft	166.2	1.18	0.127
	1|ID + Yr + BrSta + PartnStatAft + PartnStatAft*Yr	166.9	1.87	0.090
	1|ID	181.3	15.21	0.000
zl A/G diff	1|ID + Yr	170.3	0.00	0.181
	1|ID + Yr + BrSta	170.6	0.29	0.157
	**1|ID + Yr + PartnStatAft (selected model)**	**170.9**	**0.62**	**0.133**
	1|ID	177.3	7.06	0.005
zl URIC diff	1|ID + Yr	167.4	0.00	0.406
	**1|ID + Yr + PartnStatAft (selected model)**	**169.7**	**2.36**	**0.125**
	1|ID + Yr + Sex	169.9	2.52	0.115
	1|ID	177.1	9.72	0.003
zl CK diff	1|ID + Yr	148.0	0.00	0.289
	**1|ID + Yr + PartnStatAft (selected model)**	**148.8**	**0.82**	**0.192**
	1|ID + Yr + Sex	150.1	2.14	0.099
	1|ID	163.9	15.90	0.000

LEGEND: z, standardized data; l, log-transformed data; r: residuals used; TL, relative telomere length (T/S ratio); TROC, annual telomere rate of change; OHdG, plasma 8-hydroxy-2'-deoxyguanosine concentration; TBARSt, plasma thiobarbituric acid reactive substances divided by plasma triglycerides concentration; TAC, total antioxidant capacity of plasma; PI_c, peroxidation index in blood cells; PI_p, peroxidation index in plasma; $\omega6/\omega3_c$, omega-6/omega-3 ratio in blood cells; $\omega6/\omega3_p$, omega-6/omega-3 ratio in plasma; BM, body mass; BMvar: body mass variation during the breeding season; HL, heterophils:lymphocytes ratio; HCT/TP, hematocrit divided by plasma total protein concentration; GLU, plasma glucose concentration; TRIG, plasma triglycerides concentration; BHB, plasma beta-hydroxybutyrate concentration; TP, plasma total protein concentration; ALB, plasma albumin concentration; GLOB, plasma globulin concentration; A/G, albumin/globulin ratio in plasma; URIC, plasma uric acid concentration; CK, plasma creatine kinase activity.
