# Supplementary material for: Under pressure—exploring partner changes, physiological responses and telomere dynamics in northern gannets across varying breeding conditions
Source: PeerJ. 2023 Dec 1;11:e16457. doi: 10.7717/peerj.16457 (PMC10695113; doi:10.7717/peerj.16457)
Supplement: Supplemental Information 2 — All possible submodels were derived from four predictors and two interactions as fixed factors and bird ID as random factor. Model selection was based on this criterion: the most parsimonious model including partnership status, with the lowest Akaike information criterion for small samples Δ AICc < 2.5. Likelihood ratio test (using the Chi square distribution) of the best model were also used to compare the best model to a null model. Computation of p values was based on t-tests calculation using Satterthwaite approximation for the degrees of freedom. Lines with bold text are exhibit significant predictors. Yr, year; PartnStatAft, partnership status between year 1 and year 2; BrSta, breeding stage; diff, difference in biomarker results between year 1 and year 2; z, standardized data; l, log-transformed data; r: residuals used; TROC, annual telomere rate of change; OHdG, plasma 8-hydroxy-2’-deoxyguanosine concentration; TBARSt, plasma thiobarbituric acid reactive substances divided by plasma triglycerides concentration; TAC, total antioxidant capacity of plasma; PIc, peroxidation index in blood cells; PIp, peroxidation index in plasma; ω 6/ω 3c, omega-6/omega-3 ratio in blood cells; ω 6/ω 3p, omega-6/omega-3 ratio in plasma; BM, body mass; BMvar: body mass variation during the breeding season; HL, heterophils:lymphocytes ratio; HCT/TP, hematocrit divided by plasma total protein concentration; GLU, plasma glucose concentration; TRIG, plasma triglycerides concentration; BHB, plasma beta-hydroxybutyrate concentration; TP, plasma total protein concentration; ALB, plasma albumin concentration; GLOB, plasma globulin concentration; A/G, albumin/globulin ratio in plasma; URIC, plasma uric acid concentration; CK, plasma creatine kinase activity (drawing credits: David Pelletier). [file peerj-11-16457-s002.pdf]

INDEPENDENT VARIABLE	PREDICTOR	ESTIMATE ± S.E.	*t*-VALUE (*df*)	*P*-VALUE	EFFECT SIZE (Cohen's *d*)
zlTROC (R^2 = 0.34)	Intercept	-0.14 ± 0.43	-0.32 (33)	0.753	
	PartnStatRet	0.07 ± 0.27	0.24 (16)	0.812	0.12
	**BrStatInc**	**0.86 ± 0.42**	**2.06 (34)**	**0.047**	**0.70**
	**Yr2019**	**-1.89 ± 0.28**	**-6.69 (12)**	**0.000**	**-3.86**
zlOHdGdiff (R^2 = 0.10)	Intercept	-0.25 ± 0.46	-0.56 (37)	0.579	
	PartnStatRet	0.31 ± 0.43	0.72 (37)	0.478	0.24
	**Yr2019**	**0.84 ± 0.42**	**2.00 (37)**	**0.049**	**0.66**
zlTBARStdiff (R^2 = 0.13)	Intercept	1.40 ± 0.75	1.89 (42)	0.066	
	**PartnStatRet**	**-1.82 ± 0.89**	**-2.05 (42)**	**0.047**	**-0.63**
	**BrStatInc**	**-2.08 ± 0.82**	**-2.54 (42)**	**0.015**	**-0.78**
	PartnStatRet:BrStatInc	1.98 ± 0.99	1.99 (42)	0.053	0.61
zlTACdiff (R^2 = 0.54)	**Intercept**	**-1.83 ± 0.42**	**-4.34 (35)**	**0.000**	
	**PartnStatRet**	**0.92 ± 0.37**	**2.48 (35)**	**0.020**	**0.84**
	**Yr2019**	**1.59 ± 0.41**	**3.92 (35)**	**0.000**	**1.32**
	**zlURICdiff**	**0.41 ± 0.14**	**2.87 (35)**	**0.007**	**0.97**
zlPIcdiff (R^2 = 0.00)	**Intercept**	**0.55 ± 0.20**	**2.62 (37)**	**0.013**	
zlPIpdiff (R^2 = 0.40)	Intercept	1.36 ± 0.66	2.06 (11)	0.064	
	PartnStatRet	0.39 ± 0.60	0.65 (11)	0.530	0.39
	Yr2019	-0.67 ± 0.56	-1.19 (11)	0.259	-0.72
	BrStatInc	-1.26 ± 0.70	-1.82 (11)	0.096	-1.10
zlw6w3cdiff (R^2 = 0.24)	Intercept	-0.09 ± 0.41	-0.22 (36)	0.825	
	PartnStatRet	0.33 ± 0.39	0.84 (36)	0.405	0.28
	**Yr2019**	**-1.23 ± 0.38**	**-3.24 (36)**	**0.003**	**-1.08**
zlw6w3pdiff (R^2 = 0.02)	**Intercept**	**0.58 ± 0.24**	**2.43 (18)**	**0.026**	
zlHLdiff (R^2 = 0.11)	Intercept	-0.68 ± 0.48	-1.42 (46)	0.163	
	PartnStatRet	0.12 ± 0.43	0.28 (46)	0.780	0.08
	**Yr2019**	**1.08 ± 0.44**	**2.46 (46)**	**0.018**	**0.73**
rzlHCTTPdiff (R^2 = 0.42)	**Intercept**	**1.11 ± 0.53**	**2.08 (42)**	**0.043**	
	PartnStatRet	0.72 ± 0.40	1.82 (42)	0.077	0.56
	**Yr2019**	**-1.45 ± 0.42**	**-3.45 (42)**	**0.001**	**-1.06**
	**BrStatInc**	**-1.23 ± 0.48**	**-2.57 (42)**	**0.014**	**-0.79**
rzlBMdiff (R^2 = 0.16)	**Intercept**	**-0.68 ± 0.32**	**-2.13 (47)**	**0.038**	
	PartnStatRet	0.47 ± 0.25	1.89 (47)	0.064	0.55
	**BrStatInc**	**0.77 ± 0.30**	**2.57 (47)**	**0.013**	**0.75**
rzlBMVardiff (R^2 = 0.39)	**Intercept**	**-1.30 ± 0.23**	**-5.59 (23)**	**0.000**	
	**PartnStatRet**	**0.72 ± 0.19**	**3.92 (9)**	**0.004**	**2.62**
	**Yr2019**	**1.20 ± 0.17**	**6.87 (7)**	**0.000**	**5.20**
zlGLUdiff (R^2 = 0.35)	Intercept	-1.58 ± 0.57	-2.80 (43)	0.008	
	**PartnStatRet**	**-0.49 ± 0.42**	**-1.19 (43)**	**0.242**	-0.36
	**Yr2019**	**1.48 ± 0.44**	**3.34 (43)**	**0.002**	**1.02**
	**BrStatInc**	**1.03 ± 0.51**	**2.04 (43)**	**0.047**	**0.62**
rzlTRIGdiff (R^2 = 0.13)	Intercept	-0.30 ± 0.44	-0.69 (43)	0.495	
	PartnStatRet	-0.42 ± 0.35	-1.18 (43)	0.246	-0.36
	**BrStatInc**	**0.94 ± 0.42**	**2.25 (42)**	**0.029**	**0.69**
zlBHBdiff (R^2 = 0.16)	Intercept	0.22 ± 0.40	0.56 (34)	0.580	
	**Yr2019**	**-0.73 ± 0.34**	**-2.16 (15)**	**0.047**	**-1.10**
	**BrStatInc**	**0.98 ± 0.46**	**2.13 (32)**	**0.040**	**0.75**
zlTPdiff (R^2 = 0.38)	Intercept	-0.58 ± 0.53	-1.09 (42)	0.284	
	**PartnStatRet**	**-0.87 ± 0.40**	**-2.18 (42)**	**0.035**	**-0.67**
	**Yr2019**	**1.34 ± 0.42**	**3.21 (42)**	**0.002**	**0.99**
	BrStatInc	0.88 ± 0.48	1.86 (42)	0.070	0.57
zlALBdiff (R^2 = 0.17)	Intercept	0.36 ± 0.45	0.81 (43)	0.423	
	PartnStatRet	-0.67 ± 0.41	-1.63 (43)	0.111	-0.50
	**Yr2019**	**0.92 ± 0.42**	**2.22 (43)**	**0.032**	**0.68**
zlGLOBdiff (R^2 = 0.37)	Intercept	-0.75 ± 0.54	-1.37 (42)	0.177	
	**PartnStatRet**	**-0.85 ± 0.41**	**-2.10 (42)**	**0.042**	**-0.65**
	**Yr2019**	**1.37 ± 0.43**	**3.20 (42)**	**0.003**	**0.99**
	BrStatInc	0.93 ± 0.49	1.91 (42)	0.063	0.59
zlAGdiff (R^2 = 0.21)	Intercept	0.46 ± 0.48	0.95 (43)	0.349	
	PartnStatRet	0.59 ± 0.44	1.35 (43)	0.184	0.41
	**Yr2019**	**-1.29 ± 0.45**	**-2.90 (43)**	**0.006**	**-0.89**
zlURICdiff (R^2 = 0.23)	**Intercept**	**-0.97 ± 0.48**	**-2.03 (43)**	**0.049**	
	PartnStatRet	-0.17 ± 0.43	-0.39 (43)	0.698	-0.12
	**Yr2019**	**1.53 ± 0.44**	**3.48 (43)**	**0.001**	**1.06**
zlCKdiff (R^2 = 0.34)	**Intercept**	**-0.91 ± 0.38**	**-2.39 (43)**	**0.021**	
	PartnStatRet	-0.44 ± 0.35	-1.27 (43)	0.210	-0.39
	**Yr2019**	**1.53 ± 0.35**	**4.36 (43)**	**0.000**	**1.33**
