# Supplementary material for: Under pressure—exploring partner changes, physiological responses and telomere dynamics in northern gannets across varying breeding conditions
Source: PeerJ. 2023 Dec 1;11:e16457. doi: 10.7717/peerj.16457 (PMC10695113; doi:10.7717/peerj.16457)
Supplement: Supplemental Information 3 — Results were recorded during the breeding season at Parc national de l’Île-Bonaventure-et-du-Rocher-Percé (Québec, Canada). The sample used to calculate these means is composed of the 38 individuals monitored during two or three years. BM, body mass; BMvar: body mass variation during the breeding season; GLU, plasma glucose concentration; TRIG, plasma triglycerides concentration; BHB, plasma beta-hydroxybutyrate concentration; TP, plasma total protein concentration; ALB, plasma albumin concentration; GLOB, plasma globulin concentration; A/G, albumin/globulin ratio in plasma; URIC, plasma uric acid concentration; CK, plasma creatine kinase activity; HCT, hematocrit; HCT/TP, hematocrit divided by plasma total protein concentration; HL, heterophils:lymphocytes ratio; TBARSt, plasma thiobarbituric acid reactive substances divided by plasma triglycerides concentration; OHdG, plasma 8-hydroxy-2’-deoxyguanosine concentration; TAC, total antioxidant capacity of plasma; PIc, peroxidation index in blood cells; PIp, peroxidation index in plasma; ω 6/ω 3c, omega-6/omega-3 ratio in blood cells; ω 6/ω 3p, omega-6/omega-3 ratio in plasma; TL, relative telomere length (T/S ratio); TROC, annual telomere rate of change. [file peerj-11-16457-s003.pdf]

TABLE S3. Mean ± SEM of biomarkers compared between northern gannets that changed or retained their mates in 2017, 2018 and 2019. Results were recorded during the breeding season at Parc national de l'Île-Bonaventure-et-du-Rocher-Percé (Québec, Canada). The sample used to calculate these means is composed of the 38 individuals monitored during two or three years.

YEARS			2017		2018		2019	
Partnership status			Changed	Retained	Changed	Retained	Changed	Retained
BM	Body mass	g	2925 ± 25 (*n* = 4)	2913 ± 64 (*n* = 13)	2760 ± 61 (*n* = 15)	2967 ± 40 (*n* = 22)	3081 ± 59 (*n* = 15)	3047 ± 49 (*n* = 20)
BMvar	Body mass variation	$g.day^{-1}$	2.6 ± 7.0 (*n* = 4)	0.2 ± 2.7 (*n* = 13)	-14.9 ± 1.7 (*n* = 15)	-10.8 ± 1.4 (*n* = 22)	-0.7 ± 4.8 (*n* = 13)	3.0 ± 5.4 (*n* = 17)
GLU	Glucose concentration in plasma	$mmol.L^{-1}$	15.1 ± 2.7 (*n* = 4)	14.4 ± 0.4 (*n* = 13)	12.3 ± 0.3 (*n* = 14)	13.0 ± 0.3 (*n* = 21)	14.1 ± 0.3 (*n* = 14)	14.1 ± 0.3 (*n* = 17)
TRIG	Triglycerides concentration in plasma	$mmol.L^{-1}$	0.48 ± 0.14 (*n* = 4)	0.53 ± 0.07 (*n* = 13)	0.37 ± 0.04 (*n* = 14)	0.47 ± 0.03 (*n* = 21)	0.64 ± 0.07 (*n* = 14)	0.49 ± 0.03 (*n* = 17)
BHB	Beta-hydroxybutyrate concentration in plasma	$mmol.L^{-1}$	0.82 ± 0.00 (*n* = 4)	0.97 ± 0.12 (*n* = 13)	1.70 ± 0.21 (*n* = 13)	1.49 ± 0.15 (*n* = 20)	1.36 ± 0.18 (*n* = 10)	1.98 ± 0.25 (*n* = 13)
TP	Total protein concentration in plasma	$g.L^{-1}$	41.2 ± 3.7 (*n* = 4)	39.4 ± 1.2 (*n* = 13)	37.2 ± 1.2 (*n* = 14)	35.9 ± 0.8 (*n* = 21)	42.1 ± 1.4 (*n* = 14)	41.8 ± 1.1 (*n* = 17)
ALB	Albumin concentration in plasma	$g.L^{-1}$	15.7 ± 0.7 (*n* = 4)	14.8 ± 0.5 (*n* = 13)	14.7 ± 0.6 (*n* = 14)	14.8 ± 0.2 (*n* = 21)	16.7 ± 0.4 (*n* = 14)	16.4 ± 0.4 (*n* = 17)
GLOB	Globulin concentration in plasma	$g.L^{-1}$	25.5 ± 3.0 (*n* = 4)	24.7 ± 0.8 (*n* = 13)	22.5 ± 0.7 (*n* = 14)	21.1 ± 0.6 (*n* = 21)	25.4 ± 1.2 (*n* = 14)	25.4 ± 0.9 (*n* = 17)
A/G	Albumin/globulin ratio in plasma	-	0.65 ± 0.05 (*n* = 4)	0.59 ± 0.01 (*n* = 13)	0.65 ± 0.02 (*n* = 14)	0.72 ± 0.02 (*n* = 21)	0.67 ± 0.03 (*n* = 14)	0.65 ± 0.02 (*n* = 17)
URIC	Uric acid concentration in plasma	µmol.L-1	742 ± 385 (*n* = 4)	1007 ± 132 (*n* = 13)	355 ± 63 (*n* = 14)	630 ± 70 (*n* = 21)	872 ± 104 (*n* = 14)	575 ± 61 (*n* = 17)
CK	Creatine kinase activity in plasma	$U.L^{-1}$	2194 ± 149 (*n* = 4)	1777 ± 185 (*n* = 13)	1129 ± 307 (*n* = 14)	774 ± 76 (*n* = 21)	1029 ± 183 (*n* = 14)	991 ± 83 (*n* = 17)
HCT	Hematocrit	%	45.7 ± 0.2 (*n* = 4)	45.6 ± 0.8 (*n* = 13)	47.2 ± 0.6 (*n* = 16)	47.6 ± 0.4 (*n* = 22)	46.4 ± 0.8 (*n* = 15)	48.2 ± 0.5 (*n* = 20)

YEARS			2017		2018		2019	
Partnership status			Changed	Retained	Changed	Retained	Changed	Retained
HCT/TP	Hematocrit divided by plasma total protein concentration	ml erythrocytes.g protein^{-1}	11.2 ± 1.0 (*n* = 4)	11.7 ± 0.4 (*n* = 13)	12.8 ± 0.5 (*n* = 16)	13.5 ± 0.3 (*n* = 22)	11.1 ± 0.4 (*n* = 15)	11.7 ± 0.4 (*n* = 20)
HL	Heterophils:lymphocytes ratio	-	1.8 ± 0.9 (*n* = 4)	3.1 ± 0.3 (*n* = 13)	2.7 ± 0.8 (*n* = 15)	1.9 ± 0.2 (*n* = 22)	3.7 ± 1.0 (*n* = 13)	3.7 ± 0.6 (*n* = 20)
TBARS	Thiobarbituric acid reactive substances in plasma	μmol.L^{-1}	5.5 ± 1.0 (*n* = 4)	5.9 ± 0.7 (*n* = 13)	5.0 ± 0.6 (*n* = 15)	5.5 ± 0.5 (*n* = 21)	5.3 ± 0.8 (*n* = 15)	4.7 ± 0.6 (*n* = 20)
TBARSt	Thiobarbituric acid reactive substances concentration corrected for TRIG in plasma	µmol.mmol triglycerides^{-1}	11.8 ± 1.3 (*n* = 4)	12.1 ± 1.6 (*n* = 13)	15.6 ± 3.0 (*n* = 14)	12.5 ± 1.4 (*n* = 20)	9.7 ± 2.2 (*n* = 14)	9.4 ± 1.4 (*n* = 17)
OHdG	8-hydroxy-2'-deoxyguanosine concentration	ng.mL^{-1}	26.5 ± 7.2 (*n* = 4)	64.8 ± 17.7 (*n* = 13)	31.9 ± 5.2 (*n* = 13)	52.7 ± 5.7 (*n* = 20)	84.0 ± 10.7 (*n* = 11)	71.7 ± 10.4 (*n* = 15)
TAC	Total antioxidant capacity in plasma	mmol.L^{-1} Trolox	2.9 ± 0.4 (*n* = 4)	2.8 ± 0.3 (*n* = 11)	1.1 ± 0.2 (*n* = 13)	1.4 ± 0.1 (*n* = 21)	1.5 ± 0.1 (*n* = 11)	1.5 ± 0.1 (*n* = 15)
PI_c	Peroxidation index in blood cells	-	198 ± 1 (*n* = 4)	199 ± 2 (*n* = 13)	211 ± 5 (*n* = 12)	207 ± 2 (*n* = 20)	212 ± 5 (*n* = 8)	214 ± 4 (*n* = 14)
PI_p	Peroxidation index in plasma	-	192 ± 1 (*n* = 3)	167 ± 10 (*n* = 13)	201 ± 8 (*n* = 8)	202 ± 2 (*n* = 12)	200 ± 5 (*n* = 2)	197 ± 3 (*n* = 8)
$\omega6/\omega3_c$	Omega-6/omega-3 ratio in blood cells	-	0.45 ± 0.03 (*n* = 4)	0.43 ± 0.02 (*n* = 13)	0.44 ± 0.02 (*n* = 12)	0.44 ± 0.01 (*n* = 20)	0.37 ± 0.02 (*n* = 8)	0.38 ± 0.01 (*n* = 14)
$\omega6/\omega3_p$	Omega-6/omega-3 ratio in plasma	-	0.27 ± 0.00 (*n* = 3)	0.29 ± 0.05 (*n* = 13)	0.36 ± 0.01 (*n* = 8)	0.37 ± 0.03 (*n* = 12)	0.36 ± 0.01 (*n* = 2)	0.45 ± 0.04 (*n* = 8)
TL	Telomere length in blood cells (T/S ratio)	-	1.17 ± 0.16 (*n* = 4)	1.45 ± 0.16 (*n* = 12)	1.92 ± 0.23 (*n* = 15)	1.80 ± 0.17 (*n* = 22)	0.93 ± 0.17 (*n* = 14)	0.88 ± 0.06 (*n* = 20)
TROC	Annual telomere rate of change in blood cells	T/S ratio.yr^{-1}	-	-	0.90 ± 0.37 (*n* = 4)	0.22 ± 0.23 (*n* = 11)	-1.33 ± 0.32 (*n* = 14)	-1.15 ± 0.29 (*n* = 20)
